# Selectively Growing a Highly Active Interface of Mixed Nb–Rh Oxide/2D Carbon for Electrocatalytic Hydrogen Production

**DOI:** 10.1002/advs.202104706

**Published:** 2022-02-01

**Authors:** Yang Gao, Lu Qi, Feng He, Yurui Xue, Yuliang Li

**Affiliations:** ^1^ Institute of Chemistry Chinese Academy of Sciences Beijing 100190 P. R. China; ^2^ Science Center for Material Creation and Energy Conversion Institute of Frontier and Interdisciplinary Science School of Chemistry and Chemical Engineering Shandong University Jinan 250100 P. R. China; ^3^ School of Chemical Sciences, University of Chinese Academy of Sciences Beijing 100049 P. R. China

**Keywords:** 2D carbon allotrope, electrocatalysis, graphdiyne, hydrogen energy conversion, regulation of metal valence

## Abstract

Tailorable electron distribution of the active sites is widely regarded as the key issue to boost the catalytic activity and provide mechanistic insights into the structure–property–performance relationship. Here, a selective metal atom in situ growth strategy to construct highly active interface of mixed metal atom with different Nb*_y_* RhO*_x_* species on sp‐/sp^2^‐cohybridized graphdiyne (Nb*_y_* RhO*_x_*/GDY) is reported. With this innovative idea implemented, experimental results show that the asymmetric electron distribution and the variation of coordination environment of bimetallic species significantly improve the electrocatalytic activity of Nb*_y_* RhO*_x_*/GDY. Optimal hydrogen evolution reaction (HER) activity is achieved at the Nb/Rh ratio of 0.23, exhibiting excellent HER activity with the small overpotentials of 14 and 10 mV at 10 mA cm^−2^ in alkaline and neutral electrolytes. The data show the strong potential for real‐system application of such catalysts, which outperform commercial Pt/C (20 wt%). These results shown in this study represent a platform for designing novel catalytic materials by selectively introducing metal atoms on different supports, which can be used as a general method extended to other catalytic systems.

## Graphical abstract

A selective metal atom in situ growth strategy is established to grow the highly active interface of mixed metal atom oxides on sp‐/sp^2^‐cohybridized graphdiyne (GDY), realizing the asymmetric electron distribution and the variation of coordination environment of bimetallic species The Nb*_y_* RhO*_x_*/GDY shows highly active and durable hydrogen evolution reaction performances, much better than that of commercial Pt/C in a wide pH range.

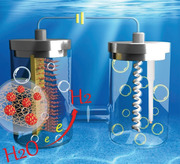

## 1 Introduction

The hydrogen evolution reaction (HER) is one of the most promising approaches for highly pure hydrogen production for fuel cells without any carbon emission.^[^ ^1^, ^2^, ^3^ ^]^ As an important class of electrocatalysts, platinum (Pt) and Pt‐based materials are still currently the benchmark electrocatalysts for HER, but their expensive features and shortages make their wide range and large use in the global energy system almost impossible.^[^ ^4^ ^]^ This is such a serious challenge for convenient, pollution‐free, and efficient use of hydrogen energy, forcing researchers to explore noble‐metal‐free or low precious metal loading electrocatalysts with high catalytic performance, low cost, and scalable synthesis. We are optimistic that various catalysts such as oxides,^[^ ^5^, ^6^ ^]^ sulfides,^[^ ^7^, ^8^ ^]^ phosphides,^[^ ^9^, ^10^ ^]^ and others can be exploited in HER. However, their energetic inefficiency and sluggish kinetics still remain difficult to apply widely in industry. Compared with traditional nanoparticles, mixed metal atom catalysts anchoring on supports with highly active interface have aroused considerable interests due to the unique characters such as the high atom utilization efficiency, large specific surface area, and extremely active and selective catalysis for various reactions.^[^ ^11^, ^12^, ^13^ ^]^ Recently, many mixed metal atom catalysts have been reported and exhibited superior catalytic performance for HER.^[^ ^11^, ^12^, ^13^, ^14^ ^]^ For example, phosphate‐substituted *β*‐NiMoO_4_ exhibits the optimal hydrogen adsorption free energy and elevated HER activity due to the abundant active electronic states.^[^ ^14^ ^]^ Ru‐Mo_2_C supported on carbon nanotube (Ru‐Mo_2_C@CNT) showed superior HER performance to commercial Pt/C in alkaline electrolyte.^[^ ^11^ ^]^ Besides, the tunable electron structures and coordination environment of the metal atom active sites can effectively tailor the catalytic performance.^[^ ^15^, ^16^, ^17^, ^18^ ^]^ Although metal atom catalysts are highly advanced in electrocatalysis, their own prominent weaknesses, such as uncontrollable size distribution, easy aggregation of active sites, and unclear valence state and structure–property–performance relationship, remain to be addressed.^[^ ^19^ ^]^

We clearly understand these defects in catalysts and try our best to explore effective strategies. It is found that the nearly perfect structure of graphdiyne (GDY) as support has the potential to overcome these issues, due to the unique and superior properties of GDY, such as sp‐ and sp^2^‐hybridized carbon network, natural pores, high intrinsic activity, and excellent stability.^[^ ^20^, ^21^, ^22^, ^23^, ^24^, ^25^, ^26^ ^]^ Previous reports have demonstrated that porous GDY could be an ideal candidate for direct use as the support and to fabricate structure controllable electrocatalysts with excellent catalytic selectivity, activity, and stability.^[^ ^27^, ^28^, ^29^, ^30^, ^31^, ^32^, ^33^, ^34^, ^35^, ^36^, ^37^, ^38^, ^39^, ^40^ ^]^ A variety of GDY‐based catalysts have been developed, e.g., those including different groups of GDY‐based zerovalent atomic catalysts,^[^ ^30^, ^31^ ^]^ heterostructured catalysts,^[^ ^32^, ^33^, ^36^, ^37^, ^39^ ^]^ quantum dot catalysts,^[^ ^27^, ^29^ ^]^ and metal‐free catalysts.^[^ ^40^ ^]^

We take the structural advantages of GDY to achieve the well‐defined, highly active interface of mixed metal atom Nb*_y_* RhO*_x_* catalysts on the surface of GDY (Nb*_y_* RhO*_x_*/GDY) through a hydrothermal reaction. The as‐synthesized Nb*_y_* RhO*_x_* species are uniformly distributed on the surface of the GDY. The optimized interface structure and strong electronic coupling effects between GDY and metal species could further enrich active sites and accelerate charge transfer to enhance the original catalytic performance. Experimental results reveal that Nb_0.23_RhO*_x_*/GDY exhibits high HER activity in alkaline and neutral conditions, with small overpotentials, ultralow Tafel slopes, large turnover frequencies (TOFs), and high long‐term stabilities, which are comparable to that of commercial Pt/C. The facile synthesis strategy coupled with high catalytic performance offers a promising electrocatalyst for hydrogen evolution.

## 2 Results and Discussion

### 2.1 Fabrication and Morphology

**Figure 1:**
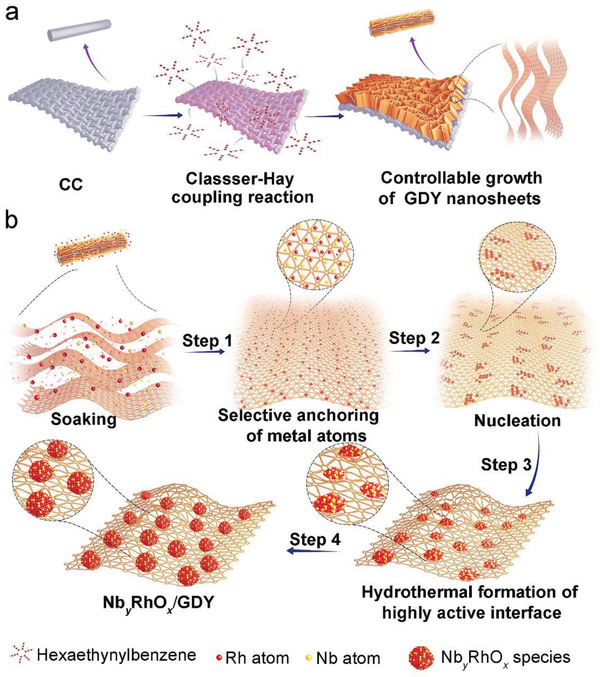
Schematic illustration of the synthesis routes for a) GDY and b) Nb*_y_* RhO*_x_*/GDY.

**Figure 2:**
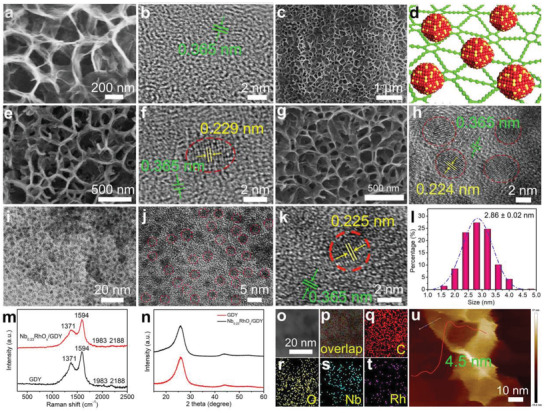
a) SEM and b) HRTEM images of GDY. c) SEM images of Nb_0.23_RhO*_x_*/GDY. d) The Nb_0.23_RhO*_x_*/GDY model. e) SEM and f) HRTEM images of NbO*_x_*/GDY. g) SEM and h) HRTEM images of RhO*_x_*/GDY. i–k) HRTEM images of Nb_0.23_RhO*_x_*/GDY. l) Size distribution of Nb_0.23_RhO*_x_* species on GDY. m) Raman spectra and n) XRD patterns of GDY and Nb_0.23_RhO*_x_*/GDY. o) Scanning TEM and p) elemental mapping images of q) C, r) O, s) Nb, and t) Rh. u) AFM image of Nb_0.23_RhO*_x_*/GDY.

**Figure** **1** illustrates the synthetic routes for Nb*_y_* RhO*_x_*/GDY samples (please see the Experimental Section for details). Typically, the self‐supported 3D GDY nanosheet arrays were grown on carbon cloth (CC) through a simple cross‐coupling reaction using hexaethynylbenzene (HEB) as precursor (Figure 1a).^[^ ^23^ ^]^ The thoroughly washed 3D GDY electrode was immersed into a mixed solution of Nb and Rh ions for selective anchoring of Nb and Rh atoms on GDY, followed by the nucleation and growth of Nb*_y_* RhO*_x_* species on GDY through a hydrothermal treatment (Figure 1b). As evidenced by high‐resolution scanning electron microscopy (SEM, **Figure** **2**a) and transmission electron microscopy (TEM, Figure 2b) images, the ultrathin GDY nanosheets exhibit a porous morphology with an interplanar spacing of 0.365 nm. After a 7 h reaction at 150 °C, Nb*_y_* RhO*_x_* species were controllably grown on GDY nanosheets (Nb*_y_* RhO*_x_*/GDY, Figure 2c,d). NbO*_x_*/GDY (Figure 2e,f) and RhO*_x_*/GDY (Figure 2g,h) were synthesized by using the same methods for references. The lattice spacings of 0.229 and 0.224 nm were assigned to the (109) plane of NbO*_x_* and (113) plane of RhO*_x_*, respectively. As shown in Figure 2i–k, the Nb_0.23_RhO*_x_* nanoparticles (NPs) were uniformly dispersed on the surface of the GDY. High‐resolution TEM (HRTEM) image (Figure 2k) clearly shows the lattice spacings of 0.365 and 0.225 nm for GDY and Nb_0.23_RhO*_x_* species in Nb_0.23_RhO*_x_*/GDY, respectively. The obtained Nb_0.23_RhO*_x_* gives a narrow size distribution histogram with the average diameter of 2.86 ± 0.02 nm (Figure 2l).

The Raman spectra of Nb_0.23_RhO*_x_*/GDY (Figure 2m) shows four peaks: at 1371 cm^−1^ (D band), 1594 cm^−1^ (G band), and the peaks at 1983 and 2188 cm^−1^ corresponding to the vibration of diyne linkage in GDY structure. The integral *I*_D_/*I*_G_ peak area ratio of Nb_0.23_RhO*_x_*/GDY was calculated to be 0.67, which is larger than that of pure GDY (0.60), indicating that more defects were generated for Nb_0.23_RhO*_x_*/GDY.^[^ ^28^, ^30^, ^31^ ^]^ The broad diffraction peaks at ≈25° and 44° arose from the GDY (Figure 2n). No diffraction peaks of Nb_0.23_RhO*_x_* were observed in the X‐ray diffraction (XRD) pattern of Nb_0.23_RhO*_x_*/GDY, which could be due to the small size and low mass loading of Nb_0.23_RhO*_x_* species on GDY (Nb and Rh weight contents are 0.0377 and 0.3094 wt%, respectively, Table S1, Supporting Information). The elemental mapping analysis reveals the uniform dispersion of C, O, Nb, and Rh elements in Nb_0.23_RhO*_x_*/GDY samples (Figure 2q–t), consistent with the X‐ray photoelectron spectroscopy (XPS) survey spectrum (Figure S1, Supporting Information). Atomic force microscopy (AFM) image in Figure 2u shows that the Nb_0.23_RhO*_x_*/GDY nanosheet has a thickness of about 4.5 nm.

### 2.2 Structure and Chemical State Analysis

**Figure 3:**
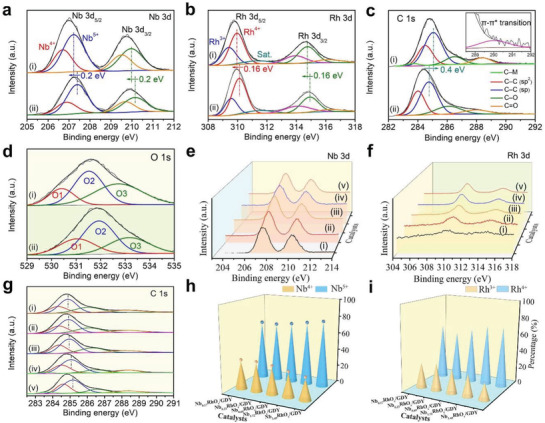
a) Nb 3d, b) Rh 3d XPS spectra of i) Nb_0.23_RhO*_x_*/GDY and ii) Nb_0.23_RhO*_x_*. c) C 1s XPS spectra of i) Nb_0.23_RhO*_x_*/GDY and ii) GDY. d) O 1s XPS spectra of i) Nb_0.23_RhO*_x_*/GDY and ii) Nb_0.23_RhO*_x_*. e) Nb 3d, f) Rh 3d, and g) C 1s XPS spectra of i) Nb_3.45_RhO*_x_*/GDY, ii) Nb_1.72_RhO*_x_*/GDY, iii) Nb_0.69_RhO*_x_*/GDY, iv) Nb_0.23_RhO*_x_*/GDY, and v) Nb_0.17_RhO*_x_*/GDY. The percentage of h) Nb^4+^ and Nb^5+^ species and i) Rh^3+^ and Rh^4+^ species in Nb*_y_* RhO*_x_*/GDY.

XPS was used to determine the composition and chemical states of the samples (Figure S1 and Table S2, Supporting Information). The 3d_5/2_/3d_3/2_ doublet peaks at 206.7/209.5 and 207.2/210.0 eV in Nb 3d XPS spectrum of Nb_0.23_RhO*_x_*/GDY (**Figure** **3**a) confirm the coexistence of Nb^4+^ and Nb^5+^ species.^[^ ^41^ ^]^ The Rh 3d XPS spectrum (Figure 3b) shows two 3d_5/2_/3d_3/2_ doublet peaks at 309.4/313.9 and 310.0/314.8 eV, which are ascribed to Rh^3+^ and Rh^4+^ species, and the corresponding satellite peaks located at 311.4 and 316.2 eV.^[^ ^42^, ^43^ ^]^ Besides, the peaks for Nb 3d and Rh 3d in Nb_0.23_RhO*_x_*/GDY showed an obvious negative shift compared to that of CC‐supported Nb_0.23_RhO*_x_*. The C 1s XPS spectrum for Nb_0.23_RhO*_x_*/GDY shows a positive shift of 0.4 eV, and two new peaks at 283.7 and 290.0 eV corresponding to the metal—C (Nb/Rh—C) interactions and *π*–π* transition, respectively, as compared to that of GDY (Figure 3c and Figure S2 (Supporting Information)).^[^ ^23^, ^44^ ^]^ Moreover, the O 1s peaks of Nb_0.23_RhO*_x_*/GDY exhibited a shift to lower binding energy compared with Nb_0.23_RhO*_x_* (Figure 3d and Figure S3 (Supporting Information)). For comparison, the C 1s and O 1s spectra of GDY and CC were also presented in Figures S2a and S3a, Supporting Information. Remarkably, with the decreasing of the Nb/Rh ratio (*R*_Nb/Rh_), the Nb 3d (Figure 3e) and Rh 3d (Figure 3f) XPS spectra for the catalysts all shift to lower binding energies, while the sp‐C peaks move to higher binding energies (Figure 3g). These findings reveal the electron‐gaining characteristics of the metal species and the electron‐donating characteristics of sp‐C in GDY. Quantitative peak deconvolution and integration of XPS analysis showed that relative contents of Nb^4+^ (Figure 3h and Figure S4 (Supporting Information)) and Rh^3+^ (Figure 3i and Figure S5 (Supporting Information)) increased with the decrease of *R*_Nb/Rh_ from 3.45 to 0.23. And Nb^4+^ and Rh^3+^ species reach the maximum contents at the *R*_Nb/Rh_ of 0.23. These findings confirm that the selective in situ growth of metal species can vary the electron structures and coordination environments of the metal active sites, and lead to the asymmetric electron distribution of catalysts, beneficial for improving the catalytic activity.

### 2.3 HER Electrocatalytic Performance

**Figure 4:**
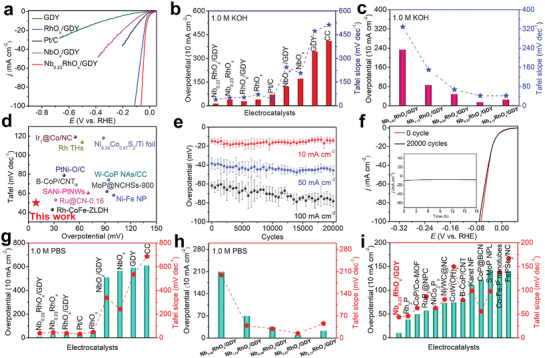
a) HER polarization curves of the samples. b,c) Overpotentials at 10 mA cm^−2^ and the Tafel slopes of the as‐prepared catalysts in 1.0 m KOH. d) Comparison of the HER performances of Nb_0.23_RhO*_x_*/GDY with the reported catalysts. e) Stability tests of Nb_0.23_RhO*_x_*/GDY in 1.0 m KOH. Error bars indicate the standard deviation of the current density. f) HER polarization curves of Nb_0.23_RhO*_x_*/GDY before and after 20 000 cycles (inset: time–current density curve of Nb_0.23_RhO*_x_*/GDY at the potential of −0.014 V vs RHE in 1.0 m KOH). g,h) Overpotentials at 10 mA cm^−2^ and Tafel slopes of the as‐synthesized catalysts in 1.0 m PBS. i) Comparison of the HER performance of the Nb_0.23_RhO*_x_*/GDY with the reported catalysts in 1.0 m PBS.

The HER performance of the samples was then studied in H_2_‐saturated 1.0 m KOH aqueous solutions. As shown in **Figure** **4**a,b, Nb_0.23_RhO*_x_*/GDY exhibits the best HER activity with the smallest overpotential (*η*_10_) of 14 mV at 10 mA cm^−2^ and Tafel slope of 42 mV dec^−1^ compared to those of NbO*_x_*/GDY (*η*_10_ = 121 mV, Tafel slope = 247 mV dec^−1^), RhO*_x_*/GDY (*η*_10_ = 28 mV, Tafel slope = 56 mV dec^−1^), Nb_0.23_RhO*_x_* (*η*_10_ = 38 mV, Tafel slope = 53 mV dec^−1^), and GDY (*η*_10_ = 344 mV, Tafel slope = 476 mV dec^−1^), respectively (Figures S6 and S7 and Table S3, Supporting Information). It was observed that the variation in *R*_Nb/Rh_ can effectively alter the HER catalytic activities, for instance, the decreasing of *R*_Nb/Rh_ from 3.45 to 0.23 leads to the obvious increase in the catalytic activity; while further decreasing in the Nb/Rh ratio to 0.17 could result in slight decrease in the HER activities (Figure 4c and Figure S8 (Supporting Information)). These values are even superior to Pt‐based materials such as commercial 20 wt% Pt/C (*η*_10_ = 71 mV; Tafel slope = 46 mV dec^−1^) and Pt clusters in hollow mesoporous carbon spheres (Pt_5_/HMCS, *η*_10_ = 46 mV, Tafel slope = 48 mV dec^−1^)^[^ ^45^ ^]^ and the reported benchmarked electrocatalysts such as Sr_2_RuO_4_ (*η*_10_ = 61 mV, Tafel slope = 51 mV dec^−1^)^[^ ^46^ ^]^ and Ni–Fe NP (*η*_10_ = 100 mV, Tafel slope = 58 mV dec^−1^)^[^ ^47^ ^]^ (Figure 4d and Table S4 (Supporting Information)). The small Tafel slope value of ≈40 mV dec^−1^ indicates that the HER on Nb_0.23_RhO*_x_*/GDY proceeds through a Volmer–Heyrovsky reaction pathway, in which the Heyrovsky step is rate‐determining.^[^ ^46^ ^]^ The durability of the Nb_0.23_RhO*_x_*/GDY was tested by accelerated cyclic voltammetry (CV) tests over 20 000 cycles (Figure 4e,f). From the results of the overpotential values recorded at 10, 50, and 100 mA cm^−2^ every 500 cycles in three independent experiments, Nb_0.23_RhO*_x_*/GDY showed high durability with a slight increase in overpotential (Figure 4e). The chronoamperometry test (*i*–*t*) at a constant overpotential of 14 mV further revealed that Nb_0.23_RhO*_x_*/GDY had robust stability with negligible losses in current density after 18 h (inset of Figure 4f).

The HER performance was next investigated in H_2_‐saturated 1.0 m phosphate‐buffered saline (PBS) solution. Nb_0.23_RhO*_x_*/GDY shows the lowest overpotential of 10 mV at 10 mA cm^−2^, outperforming commercial 20 wt% Pt/C (*η*_10_ = 42 mV), Nb_0.23_RhO*_x_* (*η*_10_ = 29 mV), RhO*_x_*/GDY (*η*_10_ = 31 mV), NbO*_x_*/GDY (*η*_10_ = 511 mV), GDY (*η*_10_ = 592 mV), and other tested catalysts, respectively (Figure 4g and Figure S9 and Table S5 (Supporting Information)). The outstanding HER activity of Nb_0.23_RhO*_x_*/GDY was further confirmed by its Tafel slope of 44 mV dec^−1^, which is very close to Pt/C (37 mV dec^−1^), RhO*_x_*/GDY (44 mV dec^−1^) and smaller than that of Nb_0.23_RhO*_x_* (51 mV dec^−1^), NbO*_x_*/GDY (381 mV dec^−1^), and GDY (603 mV dec^−1^). Moreover, a detailed catalyst activity comparison of Nb*_y_* RhO*_x_*/GDY with different Nb/Rh molar ratios was shown in Figure 4h. Nb_0.23_RhO*_x_*/GDY exhibited higher catalytic activity than Nb_3.45_RhO*_x_*/GDY (*η*_10_ = 210 mV; Tafel slope = 210 mV dec^−1^), Nb_1.72_RhO*_x_*/GDY (*η*_10_ = 70 mV; Tafel slope = 66 mV dec^−1^), Nb_0.69_RhO*_x_*/GDY (*η*_10_ = 35 mV, Tafel slope = 57 mV dec^−1^), and Nb_0.17_RhO*_x_*/GDY (*η*_10_ = 23 mV, Tafel slope = 72 mV dec^−1^). The alteration of the Nb to Rh ratio could optimize electrocatalyst activity of Nb*_y_* RhO*_x_*/GDY. Such excellent HER activity of Nb_0.23_RhO*_x_*/GDY also outperformed most of the reported precious‐metal‐based catalysts and various earth‐abundant transition metal catalysts (Figure 4i and Table S6 (Supporting Information)). Nb_0.23_RhO*_x_*/GDY exhibited almost unchanged catalytic activity after 7000 continuous CV cycles (Figure S10, Supporting Information), and a relatively small decrease in the current density after 10 h electrolysis (Figure S11, Supporting Information). SEM (Figure S12, Supporting Information) and TEM (Figure S13, Supporting Information) results revealed that the morphological and chemical structures of the catalyst were well‐preserved after continuous cycling test in 1.0 m PBS condition. Transmission electron microscopy linked with energy‐dispersive X‐ray spectroscopy (TEM–EDX) elemental mappings verified the existence of C, O, Nb, and Rh elements (Figure S14, Supporting Information).

### 2.4 The Origin of the HER Electrocatalytic Performance

**Figure 5:**
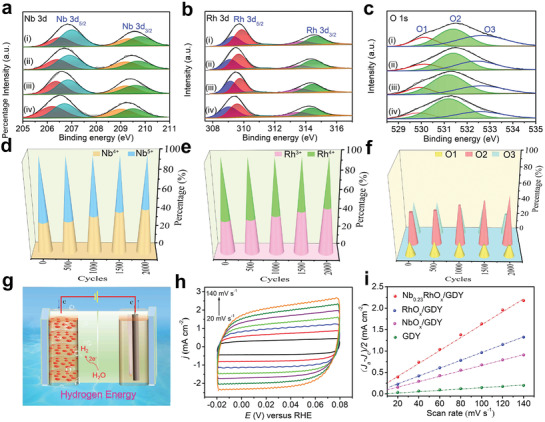
The high‐resolution a) Nb 3d, b) Rh 3d, and c) O 1s XPS spectra of the Nb_0.23_RhO*_x_*/GDY recorded at different cycles of i) 500, ii) 1000, iii) 1500, and iv) 2000 cycles during the alkaline HER. The percentage of d) Nb^4+^ and Nb^5+^ species, e) Rh^3+^ and Rh^4+^ species, and f) O1, O2, and O3 peaks in the catalyst after different cycles calculated according to (a)–(c). g) Schematic illustration of Nb_0.23_RhO*_x_*/GDY working as a cathode for efficient water reduction. h) CV measurements of Nb_0.23_RhO*_x_*/GDY at different scan rates of 20, 40, 60, 80, 100, 120, and 140 mV s^−1^ for *C*_dl_ determination in 1.0 m KOH. i) The capacitive current density for the catalysts against scan rates in 1.0 m KOH.

The electronic states and surface composition of Nb_0.23_RhO*_x_*/GDY during the HER processes were further determined by XPS measurements. The Nd 3d and Rh 3d XPS spectra for Nb_0.23_RhO*_x_*/GDY (**Figure** **5**a,b) exhibited negative shifts in binding energies as the HER electrocatalysis proceeded. According to the integrated area of peaks, the percentage of Nb^4+^ and Rh^3+^ species (Figure 5d,e) was gradually increased during repetitive potential cycling compared to the fresh catalyst. The interpretation of the O 1s spectra is actually supported by the XPS results obtained for the Nb_0.23_RhO*_x_*/GDY catalyst that underwent repetitive voltammetric scans in the HER region (Figure 5a–f). As can be seen from Figure 5c,f, the O1 peak gradually decreased and the O2 peak increased under cathodic load, which could be easily correlated with a partial reduction of M—O to M—OH. Similarly, the conversion of Nb^5+^ to Nb^4+^ (Figure 5a,d) and Rh^4+^ to Rh^3+^ (Figure 5b,e) is confirmed to take place under HER reducing conditions. Based on experimental results, Nb_0.23_RhO*_x_*/GDY showed a better catalytic activity under HER potentials than NbO*_x_*/GDY and RhO*_x_*/GDY, indicating that mixed valent Nb and Rh species were beneficial for accelerating the kinetics of water dissociation. Meanwhile, low valent Nb and Rh played an important role in enhancing catalytic activity. The catalysts Nb_0.23_RhO*_x_*/GDY working as a cathode for efficient water reduction were shown in Figure 5g. SEM (Figure S15, Supporting Information) and TEM (Figure S16, Supporting Information) analyses showed that the catalyst morphology remained almost unchanged and all Nb_0.23_RhO*_x_* species were present as individual nanocluster on GDY without any aggregation (Figure S17, Supporting Information), indicating the excellent stability of the Nb_0.23_RhO*_x_*/GDY.

The electrochemically active surface area (ECSA) of the catalysts was assessed by determining their double‐layer capacitance (*C*_dl_) (Figure 5h,i and Figure S18 (Supporting Information)). As expected, Nb_0.23_RhO*_x_*/GDY has the largest ECSA of 375 cm^2^ among those of NbO*_x_*/GDY (157.5 cm^2^), RhO*_x_*/GDY (228.8 cm^2^), GDY (34.0 cm^2^), NbO*_x_* (52.0 cm^2^), RhO*_x_* (109.0 cm^2^), Nb_0.23_RhO*_x_* (297.5 cm^2^), and other Nb*_y_* RhO*_x_*/GDY samples with different Nb to Rh ratios, which suggests the most exposed active sites of Nb_0.23_RhO*_x_*/GDY (Figures S18–S20 and Table S7, Supporting Information). Mass activity is another critical criterion to evaluate the catalytic performance of a catalyst in practical uses.^[^ ^30^ ^]^ As shown in Figure S21 (Supporting Information), the Nb_0.23_RhO*_x_*/GDY exhibited a higher mass activity than Nb_0.23_RhO_x_, RhO*_x_*/GDY, and commercial Pt/C in both alkaline and neutral conditions. The TOF value of Nb_0.23_RhO*_x_*/GDY toward HER was calculated to be 0.260 s^−1^ at the overpotential of 50 mV, which is significantly higher than those of the reference electrocatalysts such as Nb_0.23_RhO*_x_* (0.102 s^−1^), RhO*_x_*/GDY (0.210 s^−1^), and NbO*_x_*/GDY (0.027 s^−1^) (Figure S22, Supporting Information), further confirming the high catalytic activity of the mixed metal atom Nb_0.23_RhO*_x_* species on sp‐/sp^2^‐cohybridized GDY.

**Figure 6:**
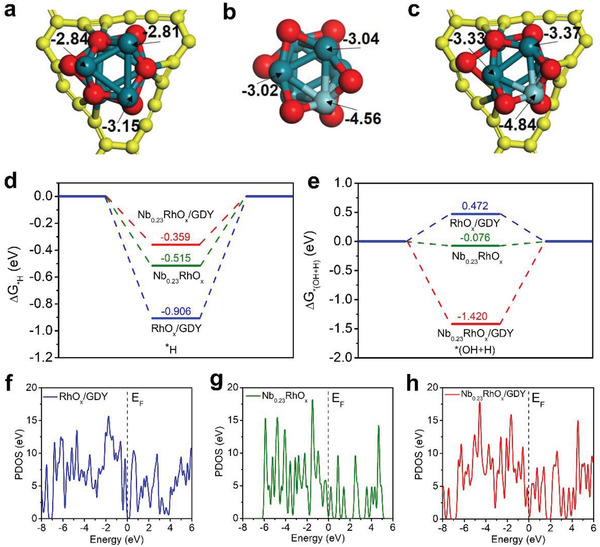
The D‐band center of Rh and Nb atoms on the surface of a) RhO*_x_*/GDY, b) Nb_0.23_RhO*_x_*, and c) Nb_0.23_RhO*_x_*/GDY, respectively. d) Gibbs free energies for H* adsorption and e) water dissociation on RhO*_x_*/GDY, Nb_0.23_RhO*_x_*, and Nb_0.23_RhO*_x_*/GDY, respectively. Total density of states (DOS) of f) RhO*_x_*/GDY, g) Nb_0.23_RhO*_x_*, h) Nb_0.23_RhO*_x_*/GDY, respectively.

To elucidate the origin of the excellent catalytic activity of Nb_0.23_RhO*_x_*/GDY, the comprehensive density functional theory (DFT) calculations were performed. It is known that the HER process can be generally described in three steps: the water activation step (H_2_O → OH* + H*, where * is the active site), the formation of the catalyst—H* intermediate, and the formation of H_2_. Based on the Tafel analysis of Nb_0.23_RhO*_x_*/GDY (42 mV dec^−1^), Nb_0.23_RhO*_x_* (53 mV dec^−1^), and RhO*_x_*/GDY (56 mV dec^−1^), it is concluded that the HER on Nb_0.23_RhO*_x_*/GDY, Nb_0.23_RhO*_x_*, and RhO*_x_*/GDY follows the Heyrovsky mechanism, i.e., H* desorption governs the reaction kinetics on the three catalysts. Hence, the free energy value of adsorbed H* (∆*G*_H*_) is the most important descriptor for HER activity.^[^ ^48^ ^]^ We first calculated the D‐band center of metal atoms on the RhO*_x_*/GDY, Nb_0.23_RhO*_x_*, and Nb_0.23_RhO*_x_*/GDY catalyst surfaces (**Figure** **6**a–c and Figure S23 (Supporting Information)). As known, the closer the D‐band center to the Fermi level, the stronger the adsorption of reaction intermediates on metal sites. It is found that the D‐band center of Rh atoms in Nb_0.23_RhO*_x_*/GDY (−3.37 eV) is much further from the Fermi Level compared to those of Rh in RhO*_x_*/GDY (−2.81 eV) and Nb_0.23_RhO*_x_* (−3.04 eV), implying that H* intermediates are less strongly bound to Nb_0.23_RhO*_x_*/GDY than to RhO*_x_*/GDY and Nb_0.23_RhO*_x_* and that their desorption is comparatively facilitated. Consequently, the Gibbs free energy of H* adsorption on Nb_0.23_RhO*_x_*/GDY is much closer to the thermoneutral state (i.e., Δ*G* = 0) relative to those of Nb_0.23_RhO*_x_* and RhO*_x_*/GDY (Figure 6d). This translates to the HER activity order of Nb_0.23_RhO*_x_*/GDY > Nb_0.23_RhO*_x_* > RhO*_x_*/GDY, which is well consistent with our experimental results. Besides, we also studied the water dissociation process, which takes place in the alkaline HER during both the Volmer and Heyrovsky steps. As shown in Figure 6e, the energy input value of Nb_0.23_RhO*_x_*/GDY for water dissociation is −1.420 eV, which is much lower than that of Nb_0.23_RhO*_x_* (−0.076 eV) and RhO*_x_*/GDY (0.472 eV), revealing the substantially promoted water dissociation ability of Nb_0.23_RhO*_x_*/GDY. The total density of states (DOS) of RhO*_x_*/GDY, Nb_0.23_RhO*_x_*, Nb_0.23_RhO*_x_*/GDY were also examined to deeply study the electronic structures (Figure 6f–h). Interestingly, all these catalysts exhibited the semiconductor properties with Dirac point. Besides, compared to Nb_0.23_RhO*_x_*, the introduction of GDY obviously broadened the energy range of orbital distribution and increased the electron density around Fermi level in Nb_0.23_RhO*_x_*/GDY. These findings solidly demonstrated that the metal‐atom‐selected aggregation of Nb_0.23_RhO*_x_* species on GDY surface played a vital role in enhancing the HER activity.

## 3 Conclusion

In summary, we have established an in situ selective growth strategy to construct a highly active interface of mixed metal atom oxides with different Nb*_y_* RhO*_x_* species on sp‐/sp^2^‐cohybridized GDY. Optimal HER activity was achieved at the Nb/Rh ratio of 0.23, outperforming that of Pt‐based electrocatalysts and other benchmarked ones. Experimental results demonstrated that selective in situ growth of metal atoms can result in an asymmetric electron distribution and high catalytic activity and stability. Selectively introducing the metal atoms on the substrates provides a new approach for the rational design and synthesis of high‐performance electrocatalysts.

## 4 Experimental Section

### Materials

Tetrabutylammonium fluoride was purchased from Alfa Aesar. HEB was brought from J&K Scientific. Toluene and tetrahydrofuran were refluxed with sodium pieces for sufficient time in order to remove the remaining water. All other reagents were purchased from Sinopharm Chemical Reagent Co., Ltd., and used without further purification unless specifically mentioned. The water used for all experiment was purified with a Millipore system. All the chemicals were of chemical grade and were used as received without further purification, weighed with MeTTLER TOLEDO electronic balance. The CC was pretreated by sonication sequentially in concentrated nitric acid, deionized water, acetone, ethanol, and deionized water before use. The copper foils could afford copper ions for the formation of copper–pyridine complexes (catalyst) for catalyzing the acetylenic coupling reaction. The freshly pretreated CC and copper foils were used immediately for the preparation of GDY.

### Preparation of GDY

Typically, GDY was synthesized as previously reported method with minor modification. Several pieces of copper foil (3 cm × 2 cm) and CC (2 cm × 2 cm) were kept at 50 °C for 2 h in a three‐necked flask containing pyridine solution (50 mL). Subsequently, 25 mg HEB was dissolved in 50 mL pyridine solution and added very slowly into the flask. The mixture was kept at 110 °C for 12 h under Ar atmosphere. The products were moved out and cleaned with hot acetone and *N*,*N*‐dimethylformamide, then thoroughly cleaned with KOH (4 m), HCl (6 m), KOH (4 m), and deionized water sequentially, and followed by drying in 40 °C vacuum oven for 12 h.

### Preparation of RhO*_x_*/GDY

Typically, 8 mg RhCl_3_·*x*H_2_O (39%) was dissolved into 15 mL of deionized water under magnetic stirring. The resulting homogeneous solutions were transferred into a 30 mL Teflon‐lined stainless‐steel autoclave. Then, a piece of GDY‐coated CC was immersed into the mixture solution and kept for 1 h. After being conducted by hydrothermal process at 150 °C for 7 h, the obtained RhO*_x_*/GDY was then thoroughly cleaned and immediately used for electrochemical tests.

### Preparation of NbO*_x_*/GDY

Typically, 2.5 mg C_4_H_4_NNbO_9_·*x*H_2_O was dissolved into 15 mL of deionized water under magnetic stirring. The resulting homogeneous solutions were transferred into a 30 mL Teflon‐lined stainless‐steel autoclave containing a piece of GDY‐coated carbon cloth and kept for 1 h. Then, the reaction was conducted under 7 h hydrothermal reaction at 150 °C. The obtained NbO*_x_*/GDY was then thoroughly cleaned and immediately used for electrochemical tests.

### Preparation of Nb*_y_* RhO*_x_*/GDY

Typically, 2.5 mg C_4_H_4_NNbO_9_·*x*H_2_O was dissolved into 10 mL of deionized water under magnetic stirring. At the same time, aqueous solution (5 mL) of RhCl_3_·*x*H_2_O (39%) with different masses (0.5, 1, 2.5, 7.5, and 10 mg) were prepared, respectively. Then, the prepared two different solutions were mixed and transferred into 30 mL Teflon‐lined stainless‐steel autoclave containing a piece of GDY‐coated carbon cloth and kept for 1 h. Then, the reaction was conducted under 7 h hydrothermal reaction at 150 °C. The final products were then thoroughly cleaned and immediately used for electrochemical tests. By varying the molar ratios of C_4_H_4_NNbO_9_·*x*H_2_O and RhCl_3_·*x*H_2_O (39%), a series of Nb*_y_* RhO*_x_*/GDY were achieved and named as Nb_3.45_RhO*_x_*/GDY, Nb_1.72_RhO*_x_*/GDY, Nb_0.69_RhO*_x_*/GDY, Nb_0.23_RhO*_x_*/GDY, and Nb_0.17_RhO*_x_*/GDY, respectively. The variation trend of the nominal and experimental Nb/Rh molar ratios of Nb*_y_* RhO*_x_*/GDY was consistent.

### Preparation of NbO*_x_*, RhO*_x_*, and Nb*_y_* RhO*_x_*

NbO*_x_*, RhO*_x_*, and Nb*_y_* RhO*_x_* were prepared according to the synthesis method of NbO*_x_*/GDY, RhO*_x_*/GDY, and Nb*_y_* RhO*_x_*/GDY, but with a minor modification, involving the replacement of the GDY‐coated carbon cloth with a bare carbon cloth.

### Characterization

SEM was recorded using an S‐4800 field emission scanning electron microscope. TEM images, HRTEM images, and elemental mapping results were obtained on a JEM‐2100F electron microscope operating at 200 kV. Atomic force microscopy (AFM, Bruker Bioscope Catalyst) was used to characterize the size and thickness of electrocatalysts. Raman spectra were measured through the Renishaw‐2000 Raman spectrometer exploiting a 473 nm excitation laser source. And a Thermo Scientific ESCALab 250Xi instrument with monochromatic Al K*α* X‐ray radiation was used to perform the XPS measurement. The powder XRD was employed with a Rigaku D/max‐2500 rotation anode X‐ray diffractometer equipped with graphite‐monochromatized Cu K*α* radiation (*λ* = 1.54178 Å) to determine the crystal structure of samples. The content of Nb and Rh elements were measured by inductive coupled plasma mass spectrometry (ICP–MS) (Thermofischer).

### ICP Measurements

First, a mixture of 6 mL HNO_3_, 1 mL HF, 1 mL HCl, and 1 mL H_2_O_2_ was transferred into a 30 mL Teflon‐lined stainless‐steel autoclave. Then, Nb_0.23_RhO*_x_*/GDY (1.2 mg cm^−2^) was immersed into the mixture solution. The mixture was kept at 180 °C for 8 h. After being cooled to room temperature, the volume of the sample solutions was increased to 25 mL by adding ultrapure water. Typically, the samples of Nb_0.23_RhO*_x_* (0.81 mg cm^−2^), RhO*_x_*/GDY (1.19 mg cm^−2^), and NbO*_x_*/GDY (1.17 mg cm^−2^) for ICP–MS tests were prepared according to the preparation method of Nb_0.23_RhO*_x_*/GDY. The values of 1.2, 0.81, 1.19, and 1.17 mg cm^−2^ for Nb_0.23_RhO*_x_*/GDY, Nb_0.23_RhO*_x_*, RhO*_x_*/GDY, and NbO*_x_*/GDY were the corresponding catalyst masses per geometric surface area, which were weighed with a MeTTLER TOLEDO electronic balance. After the ICP–MS tests, the Rh mass loadings in Nb_0.23_RhO*_x_*/GDY, Nb_0.23_RhO*_x_*, and RhO*_x_*/GDY were 3.71, 3.51, and 4.67 μg_Rh_ cm^−2^, respectively.

### Electrochemical Measurements

All electrochemical experiments were conducted through an electrochemical workstation (CHI660D, Shanghai CH. Instruments, China) with a typical three‐electrode system. The as‐prepared catalysts were used as working electrode with a geometric surface area between 2 mm × 5 mm and 5 mm × 5 mm; a graphite rod and saturated calomel electrode (SCE) were employed as the counter electrode and reference electrode, respectively. The Pt/C (20 wt%) was prepared by drop‐casting method. Typically, 1 mg of Pt/C (20 wt%) powder (Alfa Aesar) was dispersed in ethanol (950 µL) and 5 wt% Nafion solution (50 µL) under sonication for 2 h. The working electrode (Pt/C) was then prepared by drop casting 20 µL of the above solution onto the freshly cleaned GCE (*d* = 3 mm; mass loading: 4.2 μg_Pt_ cm^−2^). Before each electrochemical testing, electrolytes including 1.0 m KOH and 1.0 m PBS aqueous solutions were saturated by high‐purity H_2_ gas. The linear sweep voltammetry (LSV) polarization curves were proceeded in H_2_‐saturated electrolyte at 2 mV s^−1^ scanning rate. CV measurements were performed in an alkaline and neutral environment at 100 mV s^−1^ scanning rate in a potential range of −0.85 to −1.2 and −0.4 to −0.85 V versus SCE, respectively. The chronoamperometric test result was carried out at a constant overpotential to reach an initial current density of 10 mA cm^−2^. All potentials were converted to the reversible hydrogen electrode (vs RHE)

(1) $$E\left(\mathrm{RHE}\right)\;=\;E_{\mathrm{mea}}\;-\;iR_{s}\;+\;0.059\;\times \;\text{pH}\;+\;E^{0}\left(\mathrm{SCE}\right)$$

where *E*_mea_ is the measured potential, *i* is the current, *E* ^0^(SCE) is 0.242 V, and *R* is the Ohmic drop tested by electrochemical impedance spectroscopy.

### Calculation of ECSA

The ECSA was measured by CV within a non‐Faradaic potential range (from −0.02 to +0.08 V vs RHE) with different scan rates of 20, 40, 60, 80, 100, 120, and 140 mV s^−1^ in 1.0 m KOH. The derived *C*_dl_ was used to further assess the ECSA. The value of *C*_dl_ equaled the slope of the fitting line of $J\;=\;\frac{J_{a}\;-\;J_{c}}{2}$ against scan rates, while *J*_a_ and *J*_c_ represented the anodic and cathodic currents at 0.03 V versus SCE, respectively

(2) $$\mathrm{ECSA}\;=\;\frac{C_{\mathrm{dl}}}{C_{s}}$$

Here, the *C*_s_ is 40 µF cm^−2^ based on reported values.^[^ ^49^ ^]^

The *R*_f_ values of the catalysts were calculated as below

(3) $$R_{f}=C_{\mathrm{dl}}/C_{s}$$

### Calculation of Active Sites and TOF

The TOF values could be obtained according to the following formula

(4) $$\mathrm{TOF}\;=\;\frac{j}{2\;\times \;F\;\times \;n_{s}}\;=\;\frac{j\;\times \;N_{A}}{2\;\times \;F\;\times \;N_{s}}\;=\;\frac{j\;\times \;N_{A}}{2\;\times \;F\;\times \;N_{s,\mathrm{flat}}\;\times \;R_{f}}$$

where *j* is the current density measured at a given overpotential *η* (taken from the corresponding LSV curve), *F* is the Faraday constant (*F* = 96 485.3 C mol^−1^), *n*_s_ is the number of moles of active sites per geometric surface area, *N*_s_ is the number of active sites per geometric surface area, *N*_A_ is the Avogadro's number (*N*_A_ = 6.022 × 10^23^ mol^−1^), *R*_f_ is the roughness factor, *N*_s,flat_ is the number of surface sites per 1 cm^2^ of the flat standard electrode. According to the previous reports,^[^ ^50^ ^]^ *N*_s,flat_ was taken as 2 × 10^15^ cm^−2^ in this study.

### Computational Details

All the DFT calculations were carried out using the Vienna ab‐initio simulation package (VASP). The Perdew–Burke–Ernzerhof exchange and correlation functional was chosen.^[^ ^51^, ^52^, ^53^, ^54^ ^]^ To describe the interactions between valence electrons and ion cores, the Blöchl's all‐electron‐like projector augmented wave method was used.^[^ ^55^, ^56^ ^]^ The plane wave basis set kinetic cutoff energy of 400 eV and the *Γ*‐centered Monkhorst–Pack *k*‐point grid were applied.^[^ ^57^ ^]^ The electron occupancies were determined according to Fermi scheme with an energy smearing of 0.1 eV. The convergence tolerance of total energy calculation was determined at 1.0 × 10^−5^ eV per atom with ionic force minimization level of 0.05 eV Å^−1^. To avoid the periodic interactions, a vacuum layer as large as 20 Å was used along the *c*‐direction. The (4d, 5s, 5p), (4d, 5s, 5p), (2s, 2p), (2s, 2p), (1s) states were chosen as the valence states for Nb, Rh, C, O, and H atoms, respectively. DFT+*U* framework was imbedded within the VASP source code. The values of Hubbard *U* for Nb and Rh elements were set to be 2.00 and 2.80 eV, respectively.

To meet the experimental atomic ratio of Nb to Rh in Nb_0.23_RhO*_x_*/GDY as close as possible, a cluster of Nb_1_Rh_5_O_6_ with 12 atoms was employed to construct the quantum dot model on the graphdiyne support with 18 C atoms. The Gibbs free energy differences of intermediate species involved in the alkaline HER pathways were calculated by utilizing the computational hydrogen electrode model^[^ ^58^, ^59^ ^]^

(5) ΔG=ΔE+ΔZPE−TΔS

where Δ*E* is the energy difference of adsorption, and ΔZPE and *T*Δ*S* are the zero‐point energy correction term and the entropy correction term, respectively. The two terms were obtained by the frequency calculation at *T* = 300 K.^[^ ^60^ ^]^ The Gibbs free energy of (H^+^ + e^−^) was equivalent to the energy of 1/2*G*_H2_ in the study.

## Conflict of Interest

The authors declare no conflict of interest.

## Supporting information

Supporting Information

## Data Availability

The data that support the findings of this study are available from the corresponding author upon reasonable request.
