# Supplementary material for: Selectively Growing a Highly Active Interface of Mixed Nb–Rh Oxide/2D Carbon for Electrocatalytic Hydrogen Production
Source: Adv Sci (Weinh). 2022 Feb 1;9(10):2104706. doi: 10.1002/advs.202104706 (PMC8981907; doi:10.1002/advs.202104706)
Supplement: Supplementary file 1 — Supporting Information [file ADVS-9-2104706-s001.pdf]

ADVANCED SCIENCE
Open Access

Supporting Information

for *Adv. Sci.*, DOI 10.1002/advs.202104706

Selectively Growing a Highly Active Interface of Mixed Nb–Rh Oxide/2D Carbon for Electrocatalytic Hydrogen Production

Yang Gao, Lu Qi, Feng He, Yurui Xue and Yuliang Li**

WILEY-VCH

ADVANCED SCIENCE
Open Access

Supporting Information

for *Adv. Sci.*, DOI: 10.1002/advs.202104706

Selectively Growing a Highly Active Interface of Mixed Nb-Rh Oxide/2D Carbon for Electrocatalytic Hydrogen Production

Yang Gao, Lu Qi, Feng He, Yurui Xue, and Yuliang Li**

Supporting Information

Selectively Growing a Highly Active Interface of Mixed Nb-Rh Oxide/2D Carbon for Electrocatalytic Hydrogen Production

Yang Gao, Lu Qi, Feng He, Yurui Xue, and Yuliang Li**

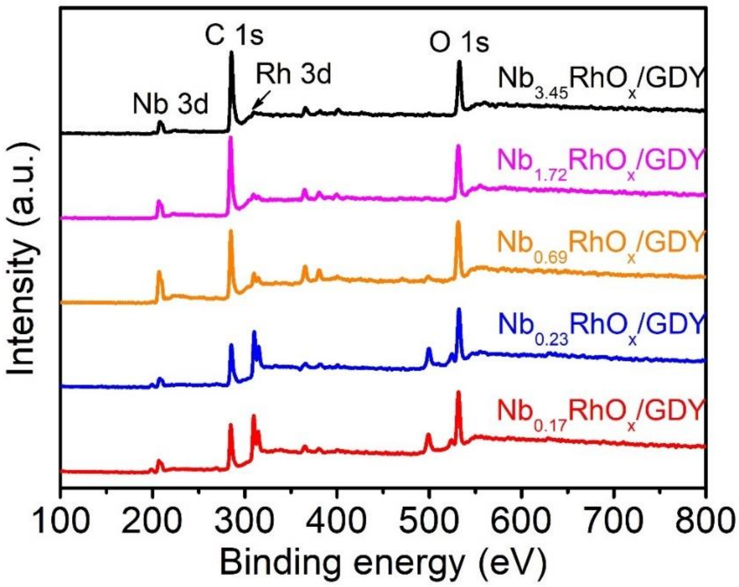

Figure S1. The XPS survey spectra of $Nb_{3.45}RhO_x$/GDY, $Nb_{1.72}RhO_x$/GDY, $Nb_{0.69}RhO_x$/GDY, $Nb_{0.23}RhO_x$/GDY and $Nb_{0.17}RhO_x$/GDY.

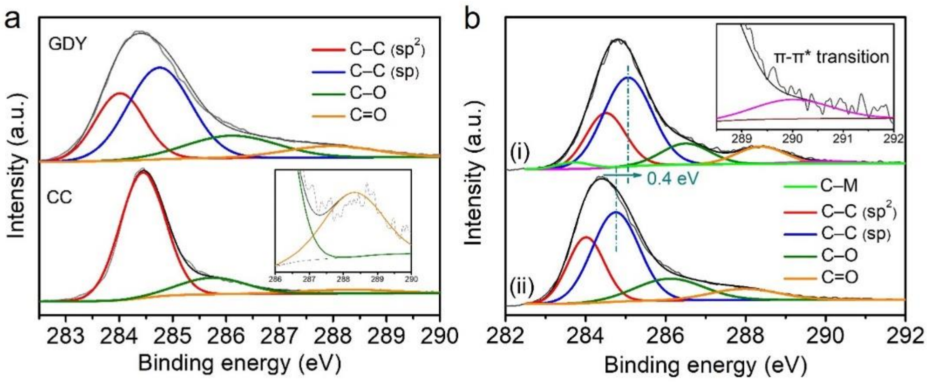

Figure S2. C 1s XPS spectra of a) GDY and CC. b) C 1s XPS spectra of (i) $Nb_{0.23}RhO_x$/GDY and (ii) $Nb_{0.23}RhO_x$.

As shown in Figure S2a, the C 1s XPS spectra of GDY can be deconvoluted into four peaks: 284.0 (sp^2–C), 284.8 (sp–C), 286.1 (C–O), and 288.0 eV (C=O), respectively. Compared with pristine GDY, the C 1s XPS spectra of CC showed three peaks: at 284.4 eV (sp^2–C), 285.7 (C–O), and 288.3 eV (C=O), respectively.

As shown in Figure S2b, the C 1s XPS spectra of $Nb_{0.23}RhO_x$/GDY were deconvoluted into six peaks: at 283.7 eV (Nb/Rh–C), 284.5 eV, (sp^2–C), 285.1 eV, (sp–C), 286.5 eV, (C–O), 288.4 (C=O) and 290.0 eV (π–π* transition). The integration area of the sp^2– and *sp*– hybridized carbon was 0.5, revealing that the integrity of the GDY structure was maintained after the hydrothermal reaction.

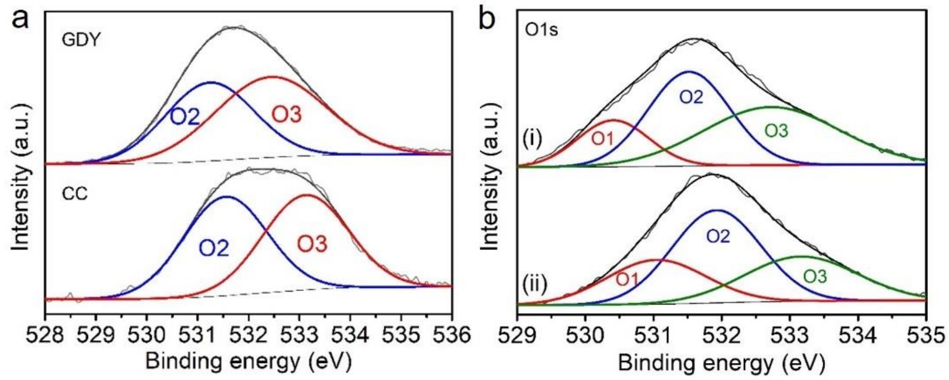

Figure S3. The high-resolution O 1s XPS spectra of a) GDY and CC. b) The high-resolution O 1s XPS spectra of (i) $Nb_{0.23}RhO_x$/GDY and (ii) $Nb_{0.23}RhO_x$.

The O 1s XPS spectra of GDY and CC (Figure S3a) show two peaks centered at 531.2-531.5 and 532.4-533.1 eV, corresponding to the adsorbed oxygen and water molecules, respectively. The O 1s spectra of $Nb_{0.23}RhO_x$/GDY (Figure S3b) could be deconvoluted into three characteristic peaks at 530.4 eV (O1 for oxygen atoms bonded to metals), 531.5 eV (O2 for surface metal hydroxides) and 532.7 eV (O3 for adsorbed water molecules), respectively.[1]

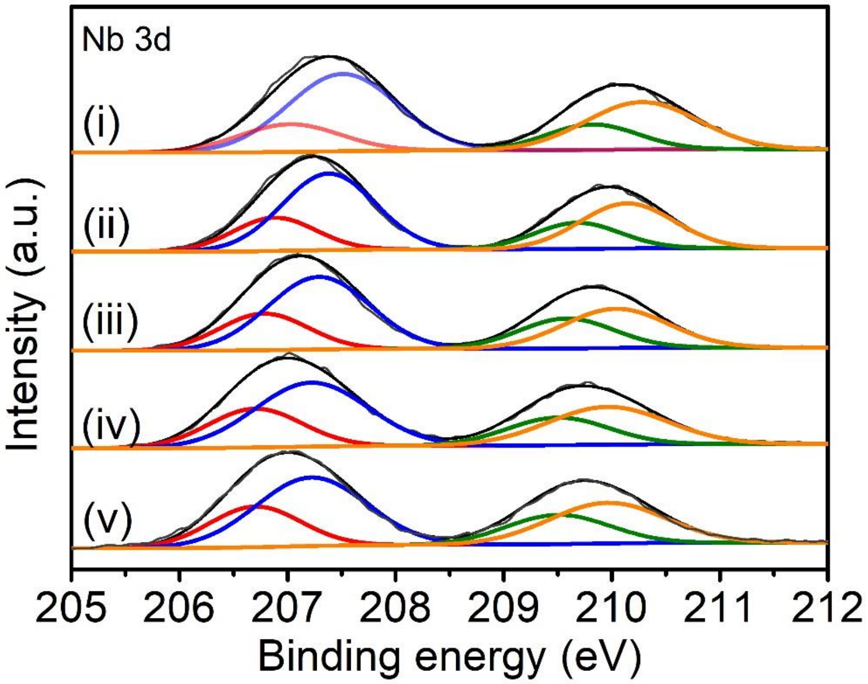

Figure S4. The high-resolution Nb 3d XPS spectra of (i) $Nb_{3.45}RhO_x$/GDY, (ii) $Nb_{1.72}RhO_x$/GDY, (iii) $Nb_{0.69}RhO_x$/GDY, (iv) $Nb_{0.23}RhO_x$/GDY and (v) $Nb_{0.17}RhO_x$/GDY.

As shown in Figure S4, the Nb 3d peaks exhibited progressive red-shifts from 207.33 eV ($Nb_{3.45}RhO_x$/GDY) to 207.19 eV ($Nb_{1.72}RhO_x$/GDY) to 207.19 eV ($Nb_{0.69}RhO_x$/GDY) to 207.01 eV ($Nb_{0.23}RhO_x$/GDY) and 207.01 eV ($Nb_{0.17}RhO_x$/GDY), respectively. Quantitative peak deconvolution and integration of XPS analysis showed that the relative content of Nb^{4+} was increased from 24.5% ($Nb_{3.45}RhO_x$/GDY) to 27.8% ($Nb_{1.72}RhO_x$/GDY) to 31.0% ($Nb_{0.69}RhO_x$/GDY) to 33.0% ($Nb_{0.23}RhO_x$/GDY) and then slightly decreased to 32.8% ($Nb_{0.17}RhO_x$/GDY).

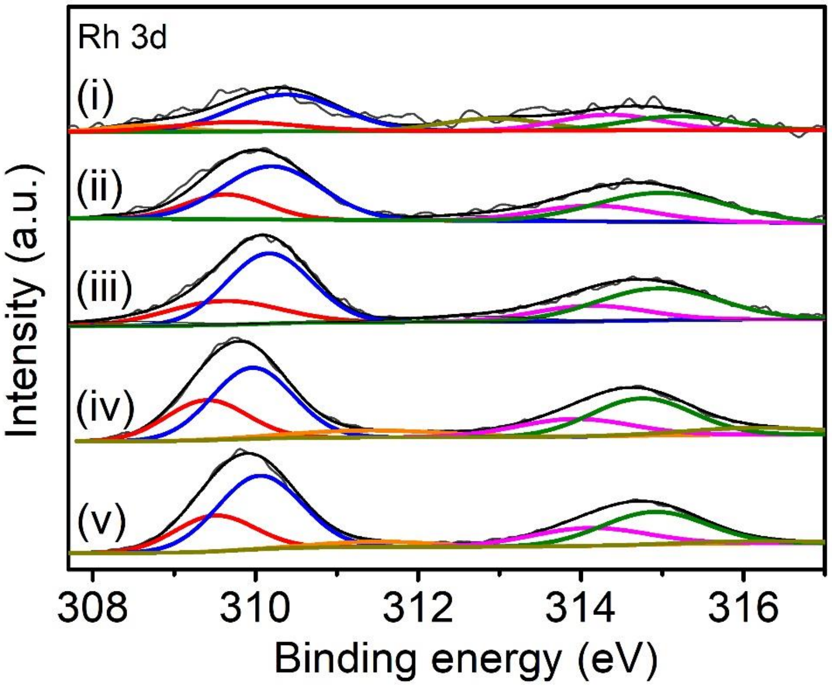

Figure S5. The high-resolution Rh 3d XPS spectra of (i) $Nb_{3.45}RhO_x$/GDY, (ii) $Nb_{1.72}RhO_x$/GDY, (iii) $Nb_{0.69}RhO_x$/GDY, (iv) $Nb_{0.23}RhO_x$/GDY and $Nb_{0.17}RhO_x$/GDY.

As shown in Figure S5, the Rh 3d peaks exhibited red-shifting from 310.18 eV ($Nb_{3.45}RhO_x$/GDY) to 309.99 eV ($Nb_{1.72}RhO_x$/GDY) to 309.96 eV ($Nb_{0.69}RhO_x$/GDY) to 309.76 eV ($Nb_{0.23}RhO_x$/GDY) and then a slight blue-shift to 309.85 eV ($Nb_{0.17}RhO_x$/GDY), respectively. Quantitative peak deconvolution and integration of XPS analysis showed that the content of Rh^{3+} was increased from 20.2% ($Nb_{3.45}RhO_x$/GDY) to 27.2% ($Nb_{1.72}RhO_x$/GDY) to 29.3% ($Nb_{0.69}RhO_x$/GDY) to 31.9% ($Nb_{0.23}RhO_x$/GDY) and then decreased to 29.6% ($Nb_{0.17}RhO_x$/GDY).

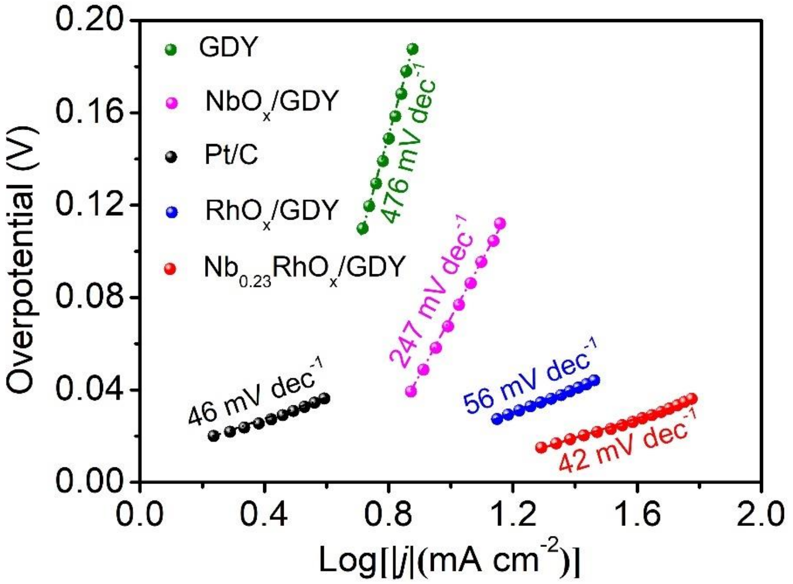

Figure S6. Tafel slopes calculated of the catalysts for HER in 1.0 M KOH.

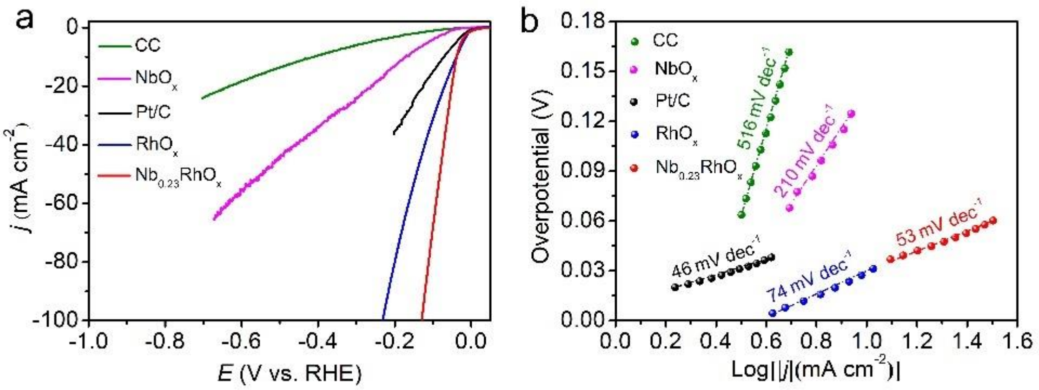

Figure S7. a) Polarization curves and b) corresponding Tafel slopes calculated of the catalysts for HER in 1.0 M KOH.

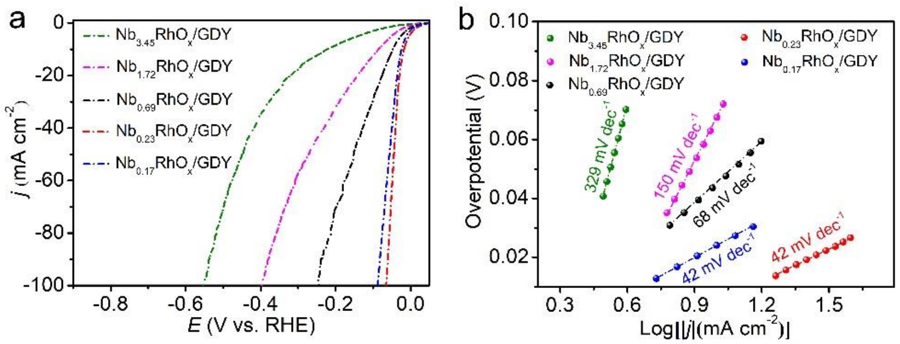

Figure S8. a) Polarization curves and b) corresponding Tafel slopes calculated of the catalysts for HER in 1.0 M KOH.

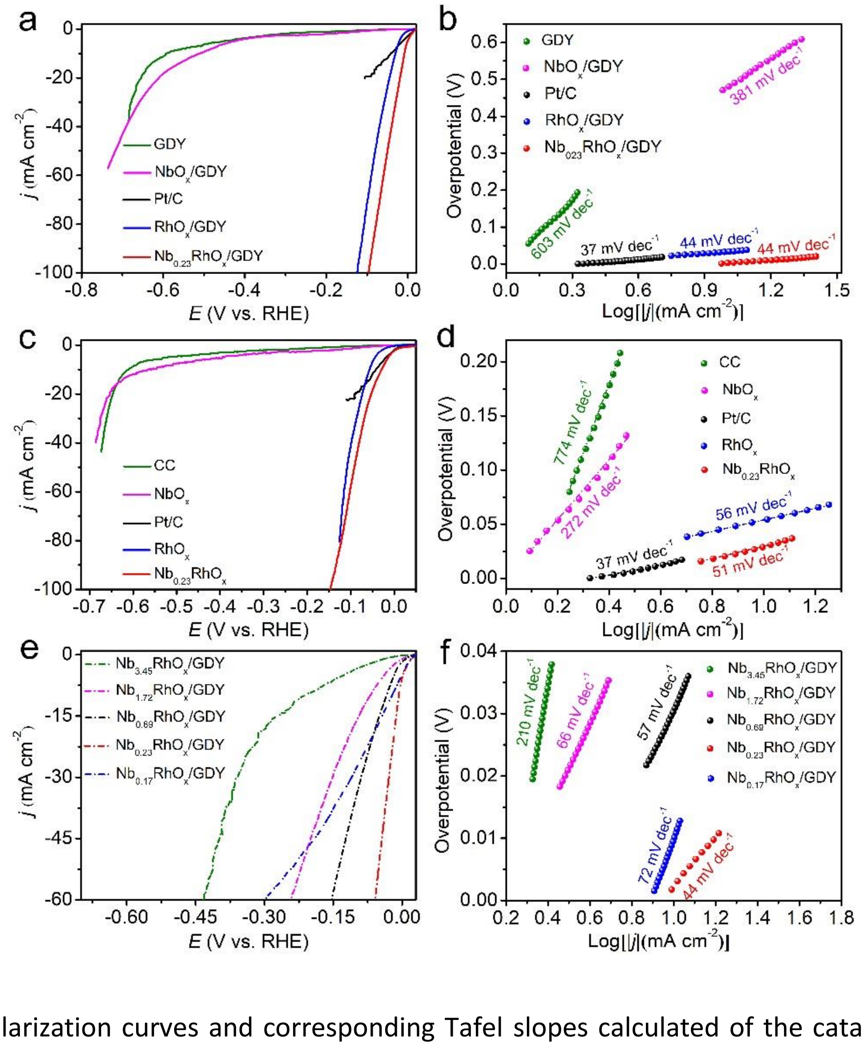

Figure S9. Polarization curves and corresponding Tafel slopes calculated of the catalysts for HER in 1.0 M PBS.

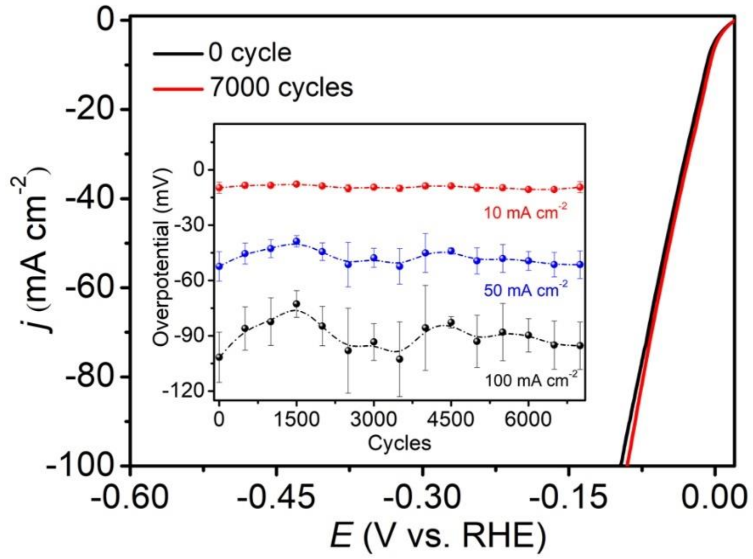

Figure S10. HER polarization curves of $Nb_{0.23}RhO_x$/GDY recorded before and after 7000 cycles (inset: Stability measurements of $Nb_{0.23}RhO_x$/GDY in 1.0 M PBS at 10, 50, and 100 mA cm^{-2} over 7000 cycles. The error bars show the standard deviation of the measurements).

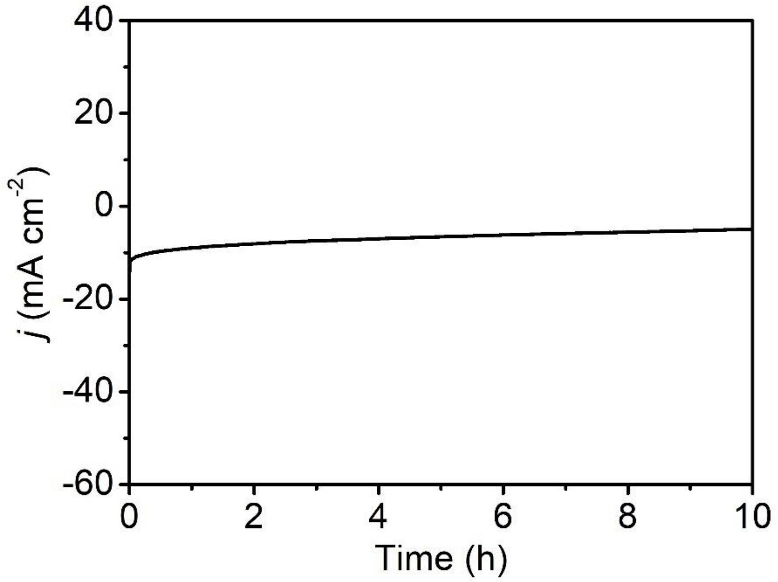

Figure S11. Time-dependent current density curve of $Nb_{0.23}RhO_x$/GDY at the potential of -0.010V (vs. RHE) in 1.0 M PBS.

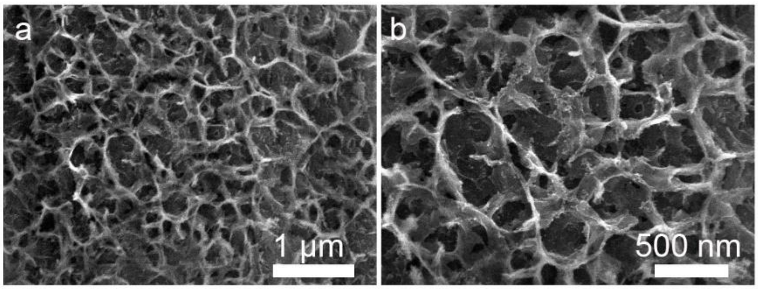

Figure S12. SEM images of $Nb_{0.23}RhO_x$/GDY after continuous cycling test in 1.0 M PBS condition.

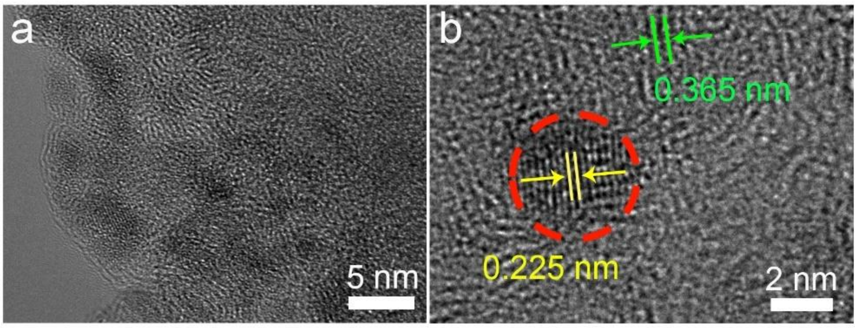

Figure S13. TEM images of $Nb_{0.23}RhO_x$/GDY after continuous cycling test in 1.0 M PBS condition.

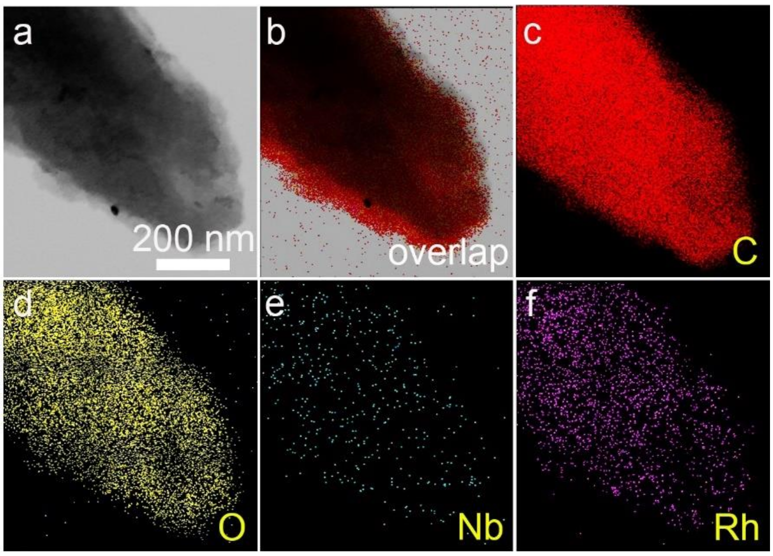

Figure S14. a) Scanning TEM image of $Nb_{0.23}RhO_x$/GDY and corresponding elemental mapping images of c) C, d) O, e) Nb and f) Rh after continuous cycling test in 1.0 M PBS condition.

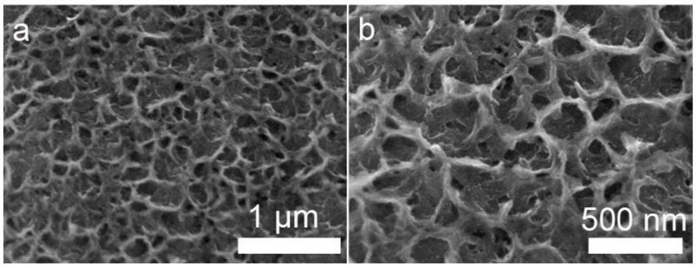

Figure S15. SEM images of $Nb_{0.23}RhO_x$/GDY after continuous cycling test in 1.0 M KOH condition.

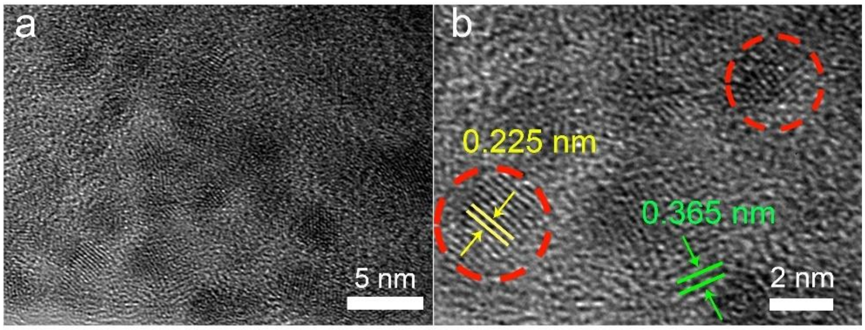

Figure S16. TEM images of $Nb_{0.23}RhO_x$/GDY after continuous cycling test in 1.0 M KOH condition.

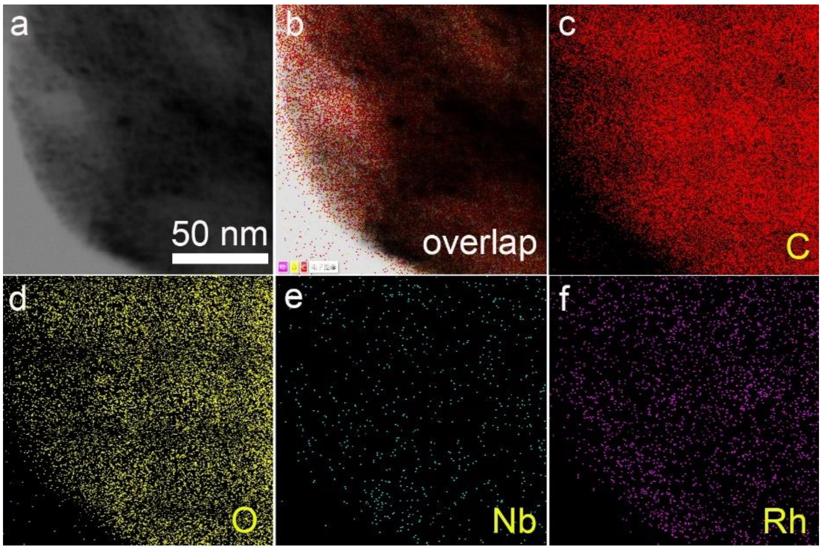

Figure S17. a) Scanning TEM image of $Nb_{0.23}RhO_x$/GDY and corresponding elemental mapping images of c) C, d) O, e) Nb and f) Rh after continuous cycling test in 1.0 M KOH condition.

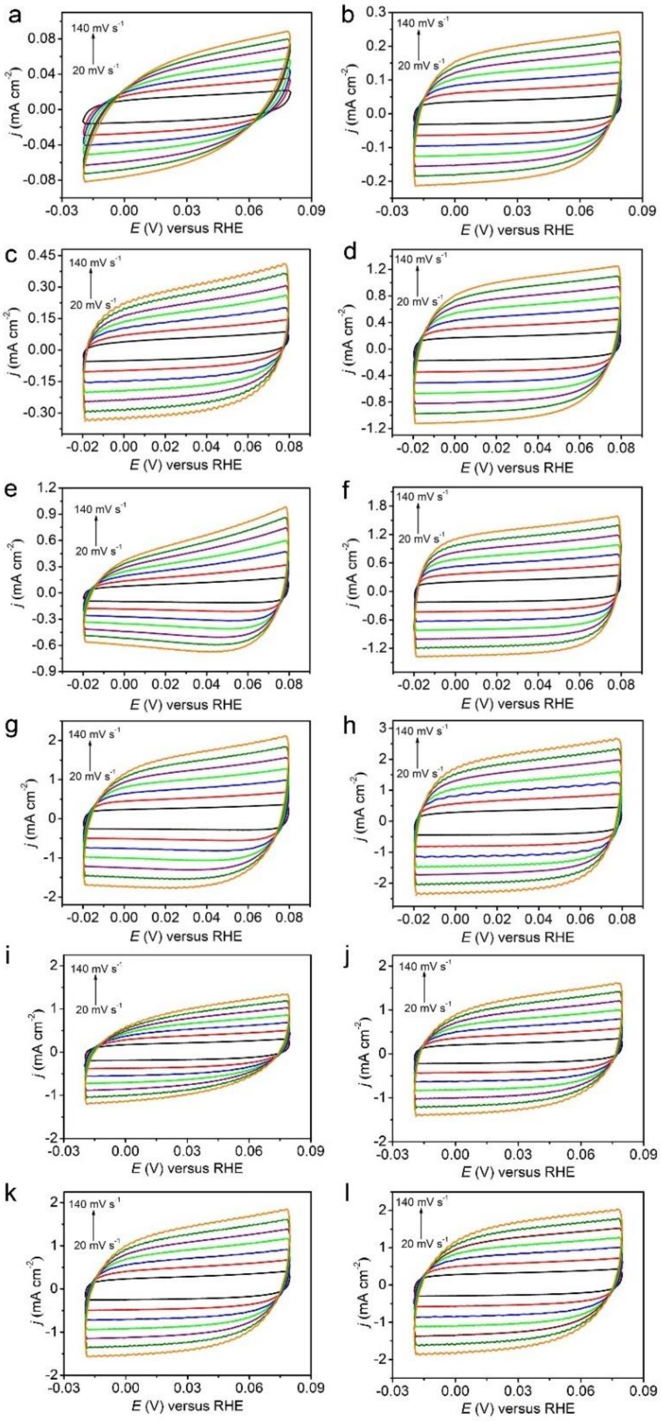

Figure S18. CV measurements of a) CC, b) GDY, c) NbO_x, d) NbO_x/GDY, e) RhO_x, f) RhO_x/GDY, g) $Nb_{0.23}RhO_x$, h) $Nb_{0.23}RhO_x$/GDY, i) $Nb_{3.45}RhO_x$/GDY, j) $Nb_{1.72}RhO_x$/GDY, k) $Nb_{0.69}RhO_x$/GDY and l) $Nb_{0.17}RhO_x$/GDY at different scan rates of 20, 40, 60, 80, 100, 120 and 140 mV s^{-1} for C_{dl} determination in 1.0 M KOH.

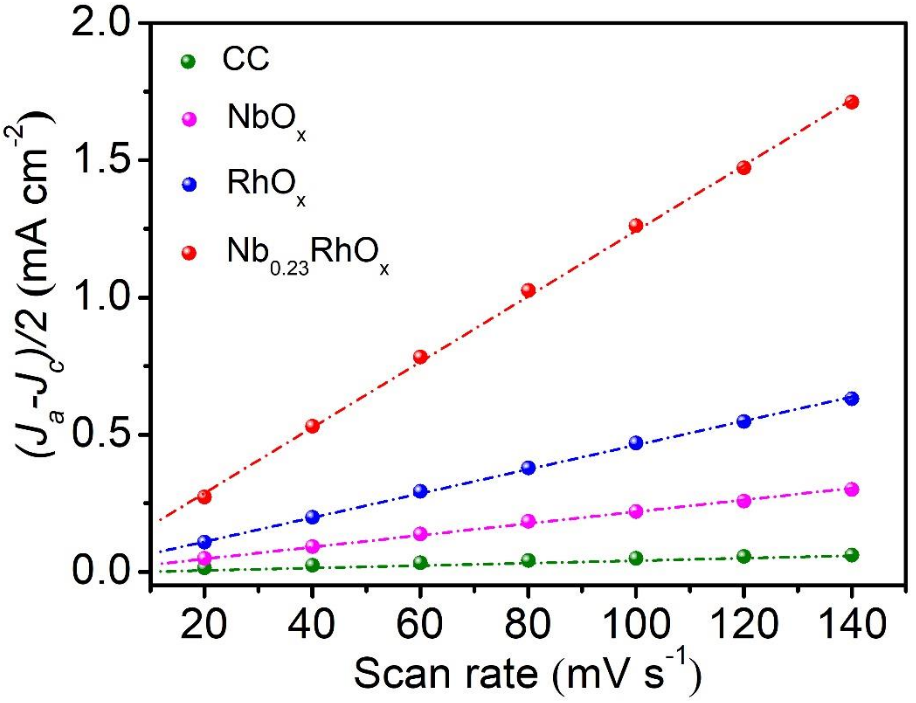

Figure S19. The capacitive current density for the catalysts against scan rates in 1.0 M KOH.

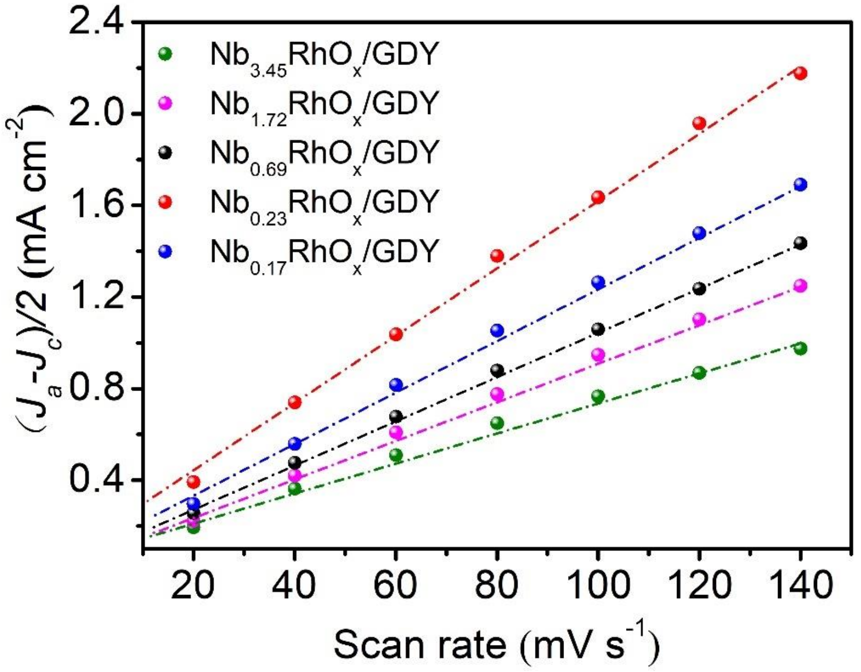

Figure S20. The capacitive current density for the catalysts against scan rates in 1.0 M KOH.

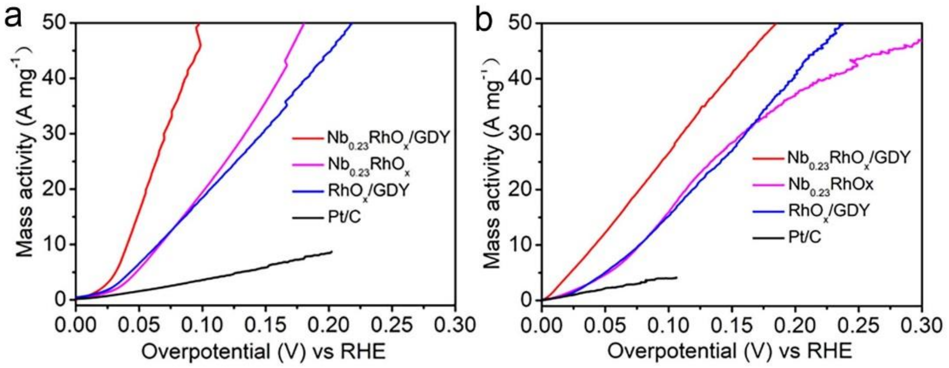

Figure S21. Mass activities of $Nb_{0.23}RhO_x$/GDY, $Nb_{0.23}RhO_x$, RhO_x/GDY and Pt/C in a) 1.0 M KOH and b) 1.0 M PBS, respectively.

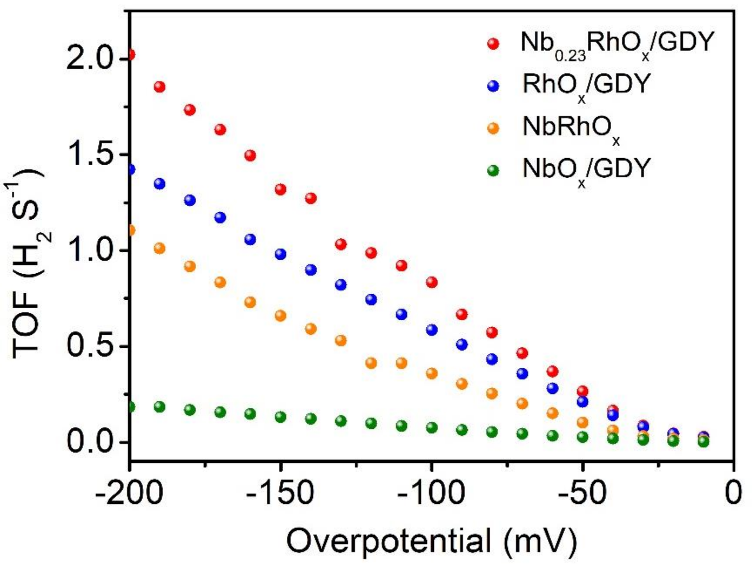

Figure S22. The TOF values of $Nb_{0.23}RhO_x$/GDY, $Nb_{0.23}RhO_x$, RhO_x/GDY and NbO_x/GDY as a function of overpotential.

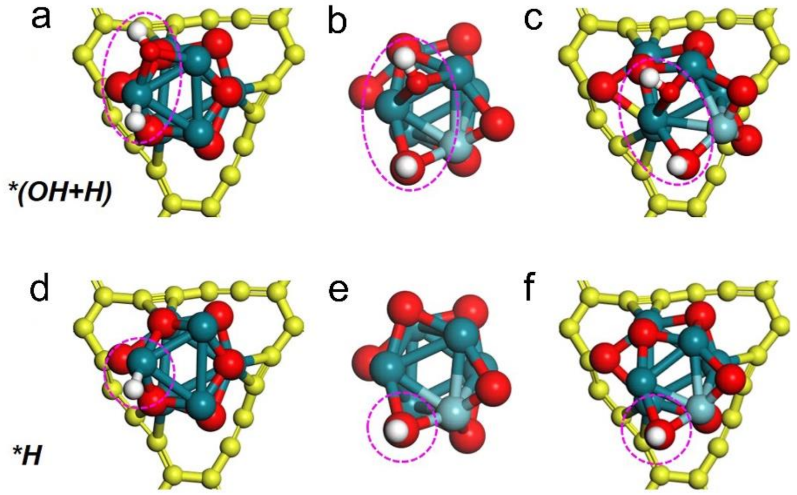

Figure S23. Top view of adsorption configurations of *(OH+H) and *H intermediates on catalysts surface of a, d) RhO_x/GDY, b, e) $Nb_{0.23}RhO_x$ and c, f) $Nb_{0.23}RhO_x$/GDY during alkaline hydrogen evolution. Yellow, blue, fluorescent, red and white balls represent C, Rh, Nb, O, and H atoms, respectively.

Table S1. The metal contents of $Nb_{0.23}RhO_x$/GDY, $Nb_{0.23}RhO_x$, NbO_x/GDY and RhO_x/GDY based on ICP analysis.

Catalyst	Nb (wt%)	Rh (wt%)
$Nb_{0.23}RhO_x$/GDY	0.0377	0.3094
$Nb_{0.23}RhO_x$	0.0023	0.4331
NbO_x/GDY	0.0210	
RhO_x/GDY		0.3921

Table S2. The Nb, Rh and O atomic contents based on the XPS spectra of Nb_yRhO_x/GDY.

		Nb atomic	Rh atomic	O atomic
Contents	$Nb_{3.45}RhO_x$/GDY	1.41%	0.21%	22.37%
	$Nb_{1.72}RhO_x$/GDY	2.18%	0.48%	24%
	$Nb_{0.69}RhO_x$/GDY	4.11%	0.95%	27.67%
	$Nb_{0.23}RhO_x$/GDY	1.76%	5.3%	32.07%
	$Nb_{0.17}RhO_x$/GDY	2.13%	4.71%	33.91%

Table S3. The overpotentials of all catalyst in 1.0 M KOH solution to achieve the current density of 10 mA cm^{-2} and 50 mA cm^{-2}, respectively.

**Catalyst**	***η* at *j* = 10 mA cm^{-2}**	***η* at *j* = 50 mA cm^{-2}**
CC	411 mV	–
GDY	344 mV	
Pt/C	71 mV	–
NbO_x	172 mV	545 mV
NbO_x/GDY	121 mV	399 mV
RhO_x	40 mV	143 mV
RhO_x/GDY	28 mV	68 mV
$Nb_{0.23}RhO_x$	38 mV	82 mV
$Nb_{3.45}RhO_x$/GDY	223 mV	449 mV
$Nb_{1.72}RhO_x$/GDY	86 mV	272 mV
$Nb_{0.69}RhO_x$/GDY	48 mV	153 mV
$Nb_{0.23}RhO_x$/GDY	14 mV	42 mV
$Nb_{0.17}RhO_x$/GDY	25 mV	56 mV

Tabel S4. Comparison of overpotential (η) at current density of 10 mA cm^{-2} and Tafel slopes of $Nb_{0.23}RhO_x$/GDY with recently reported catalysts in 1.0 M KOH aqueous solution.

**Catalysts**	**η at j = 10 mA cm^{-2} (mV)**	**Tafel slope**	
**$Nb_{0.23}RhO_x$/GDY**	**14**	**42**	**This work**
Pt_5/HMCS	46	48	*Adv. Mater*. **2020**, *32*, 1901349
RhPd-H	40	36	*J. Am. Chem. Soc*. **2020**, *142*, 3645.
Rh-doped CoFe-ZLDH	28	43	*Adv. Funct. Mater*. **2020**, *30*, 2003556.
Rh-doped CoFe-LDH	75	64	
Pt1/N-C	46	37	*Nat. Commun*. **2020**, *11*, 1029.
Ir-NSG	18.5	28	*Nat. Commun*. **2020**, *11*, 4246.
C-$Ni_{1-x}O$	27	36	*Nat. Commun*. **2020**, *11*, 590.
$NiMoO_x$/NiMoS	38	38	*Nat. Commun*. **2020**, *11*, 5462.
CoNC@Co_2N	62	—	*Adv. Energy Mater*. **2020**, 2002214
B-CoP/CNT	56	69	*Angew. Chem. Int. Ed*. **2020**, *59*, 4154.
Sr_2RuO_4	61	51	*Nat. Commun*. **2019**, *10*, 149.
CoP/Co-MOF	34	56	*Angew. Chem*. **2019**, *131*, 4727.
Ni-Fe NP	100	58	*Nat. Commun*. **2019**, *10*, 5599.
$NiMoO_x$-$Ni(OH)_2$/NF	36	38	*Adv. Energy Mater*. **2019**, *9*, 1902703.
MoP@NCHSs-900	92	62	*Angew. Chem. Int. Ed*. **2019**, *58*, 327.

W-CoP NAs/CC	94	74	*Small*. **2019**, *15*, 1902613
Mo_2N-Mo_2C/HGr	154	152	*Adv. Mater*. **2018**, *30*, 1704156.
PtNi-O/C	39.8	79	*J. Am. Chem. Soc*. **2018**, *140*, 9046.
Ni@Ni_2P-Ru	31	41	*J. Am. Chem. Soc*. **2018**, *140*, 2731.
Rh_2P	30	50	*Adv. Energy Mater*. **2018**, *8*, 1703489.
FeCoNi-HNTAs	58	38	*Nat. Commun*. **2018**, *9*, 2452.
Ru@CN-0.16	32	53	*Energy Environ. Sci*. **2018**, *11*, 800.
Pt-$Co(OH)_2$/C	32	70	*ACS Catal*. **2017**, *7*, 7131.
Rh THs	63	114	*Chem. Mater*. **2017**, *29*, 5009.
Pt_2Ni_2 NWs-S/C	42	—	*Nat. Commun*. **2017**, *8*, 14580.
RuCo@NC	28	31	*Nat. Commun*. **2017**, *8*, 14969.
CoP@BCN	122	59	*Adv. Energy Mater*. **2017**, *7*, 1601671.
c-$CoSe_2$/CC	190	85	*Adv. Mater*. **2016**, *28*, 7527.
NF-Ni_3Se_2/Ni	203	79	*Nano Energy*. **2016**, *24*, 103.
NiCoP/rGO	209	124	*Adv. Funct. Mater*. **2016**, *26*, 6785.
$Ni_{0.33}Co_{0.67}S_2$/Ti foil	88	118	*Adv. Energy Mater*. **2015**, *5*, 1402031.

Table S5. The overpotentials of all catalysts in 1.0 M PBS solution to achieve the current density of 10 mA cm^{-2} and 50 mA cm^{-2}, respectively.

**Catalysts**	***η* at *j* =10 mA cm^{-2} (mV)**	***η* at *j* = 50 mA cm^{-2}**
CC	611 mV	–
GDY	592 mV	–
Pt/C	42 mV	–
NbO_x	566 mV	–
NbO_x/GDY	511 mV	–
RhO_x	57 mV	108 mV
RhO_x/GDY	31 mV	81 mV
$Nb_{0.23}RhO_x$	29 mV	92 mV
$Nb_{3.45}RhO_x$/GDY	210 mV	413 mV
$Nb_{1.72}RhO_x$/GDY	70 mV	214 mV
$Nb_{0.69}RhO_x$/GDY	35 mV	134 mV
$Nb_{0.23}RhO_x$/GDY	10 mV	53 mV
$Nb_{0.17}RhO_x$/GDY	23 mV	226 mV

Tabel S6. Comparison of overpotential (η) at current density of 10 mA cm^{-2} and Tafel slopes of $Nb_{0.23}RhO_x$/GDY with recently reported catalysts in 1.0 M PBS aqueous solution.

Catalysts	η at j = 10 mA cm^{-2} (mV)	Tafel slope (mV dec^{-1})	Ref.
**$Nb_{0.23}RhO_x$/GDY**	**10**	**44**	**This work**
B-CoP/CNT	79	80	*Angew. Chem. Int. Ed*. **2020**, *59*, 4154.
Karst NF	110	99	*Energy Environ. Sci*. **2020**, *13*, 174.
Ir-NSG	22	21.2	*Nat. Commun*. **2020**, *11*, 4246.
CoP/Co-MOF	49	63	*Angew. Chem*. **2019**, *131*, 4727.
S-MoP NPL	142	98	*ACS Catal*. **2019**, *9*, 651.
Co-Fe-P nanotubes	138	138	*Nano Energy*. **2019**, *56*, 225.
$FePSe_3$/NC	140	167	*Nano Energy*. **2019**, *57*, 222.
NiCoP NS/NF	170	—	*J. Am. Chem. Soc*. **2018**, *140*, 5241.
Rh_2P	38	46	*Adv. Energy Mater*. **2018**, *8*, 1703489.
Ni/WC@NC	73	80.9	*Energy Environ. Sci*. **2018**, *11*, 2114.
Ru@CN-0.16	ca. 100	N/A	*Energy Environ. Sci*. **2018**, *11*, 800.
Ni_2P@NPCNFs	185	203	*Angew. Chem. Int. Ed*. **2018**, *57*, 1963.
$CoW(OH)_x$	74	150	*ACS Catal*. **2018**, *8*, 5200.
$NiCo_2P_x$	63	63.3	*Adv. Mater*. **2017**, *29*, 1605502.

RuP_2@NPC	57	87	*Angew. Chem. Int. Ed*. **2017**, *129*, 11717.
S-$NiFe_2O_4$/NF	197	81	*Nano Energy*. **2017**, *40*, 264.
CoP@BCN	122	56	*Adv. Energy Mater*. **2017**, *9*, 1601671.
CoO/$CoSe_2$	337	131	*Adv. Sci*. **2016**, *3*, 1500426.
S-$NiFe_2O_4$/NF	197	81	*Angew. Chem. Int. Ed*. **2016**, *55*, 2488.

Table S7. The C_{dl}, ECSA and R_f value of catalysts CC, GDY, NbO_x, NbO_x/GDY, RhO_x, RhO_x/GDY, $Nb_{0.23}RhO_x$ $Nb_{0.23}RhO_x$/GDY and other Nb_yRhO_x/GDY in 1.0 M KOH.

Catalysts	C_{dl} (mF cm^{-2})	ECSA (cm^2)	R_f
CC	0.39	9.75	9.75
GDY	1.36	34.0	34.0
NbO_x	2.08	52.0	52.0
NbO_x/GDY	6.30	157.5	157.5
RhO_x	4.36	109.0	109.0
RhO_x/GDY	9.15	228.8	228.8
$Nb_{0.23}RhO_x$	11.9	297.5	297.5
$Nb_{0.23}RhO_x$/GDY	15.0	375.0	375.0
$Nb_{3.45}RhO_x$/GDY	6.07	151.8	151.8
$Nb_{1.72}RhO_x$/GDY	8.28	207.0	207.0
$Nb_{0.69}RhO_x$/GDY	9.71	242.8	242.8
$Nb_{0.17}RhO_x$/GDY	11.5	287.5	287.5

Reference

[1] J. N. Tiwari, N. K. Dang, S. Sultan, P. Thangavel, H. Y. Jeong, K. S. Kim, *Nat. Sustain*. **2020**, *3*, 556.
